# Supplementary material for: High-efficiency electroporation of chytrid fungi
Source: Sci Rep. 2020 Sep 15;10:15145. doi: 10.1038/s41598-020-71618-2 (PMC7493940; doi:10.1038/s41598-020-71618-2)
Supplement: Supplementary file 1 — Supplementary information. [file 41598_2020_71618_MOESM1_ESM.pdf]

High-Efficiency Electroporation of Chytrid Fungi

Andrew J.M. Swafford[1]
Shane P. Hussey[1]
Lillian K. Fritz-Laylin[1]*

[1]University of Massachusetts Amherst, Department of Biology, Amherst, MA 01003

*Corresponding author: lfritzlaylin@umass.edu

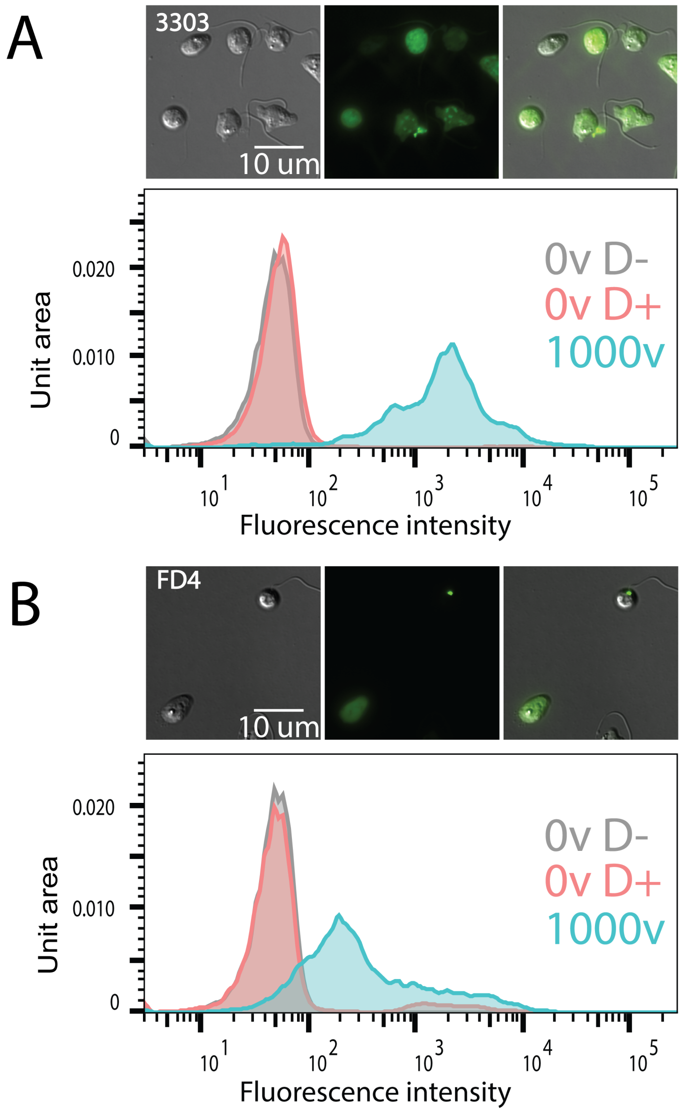

Supplemental Figure 1. This protocol is optimized for a single dextran manufacturer. **A)** Representative images and flow cytometry data for a single replicate using Thermo-Fisher Invitrogen dextran (Cat. no. 3303), the manufacturer used throughout the rest of this paper. Clearly positive cells can be observed via microscopy with dextran distributed evenly throughout the cell. **B)** Representative images and flow cytometry data for a single replicate in the same experiment as **(A)** using Sigma dextran (Cat. no. FD4). While positive cells can be observed by microscopy (bottom cell), the majority of cells appear with dextran staining restricted to a small point within the cell as seen in the top cell. Difference is also observed in the flow cytometry data, showing the 1000v, D+ experimental population with left-shifted fluorescence intensity relative to the Invitrogen 3303 dextran.

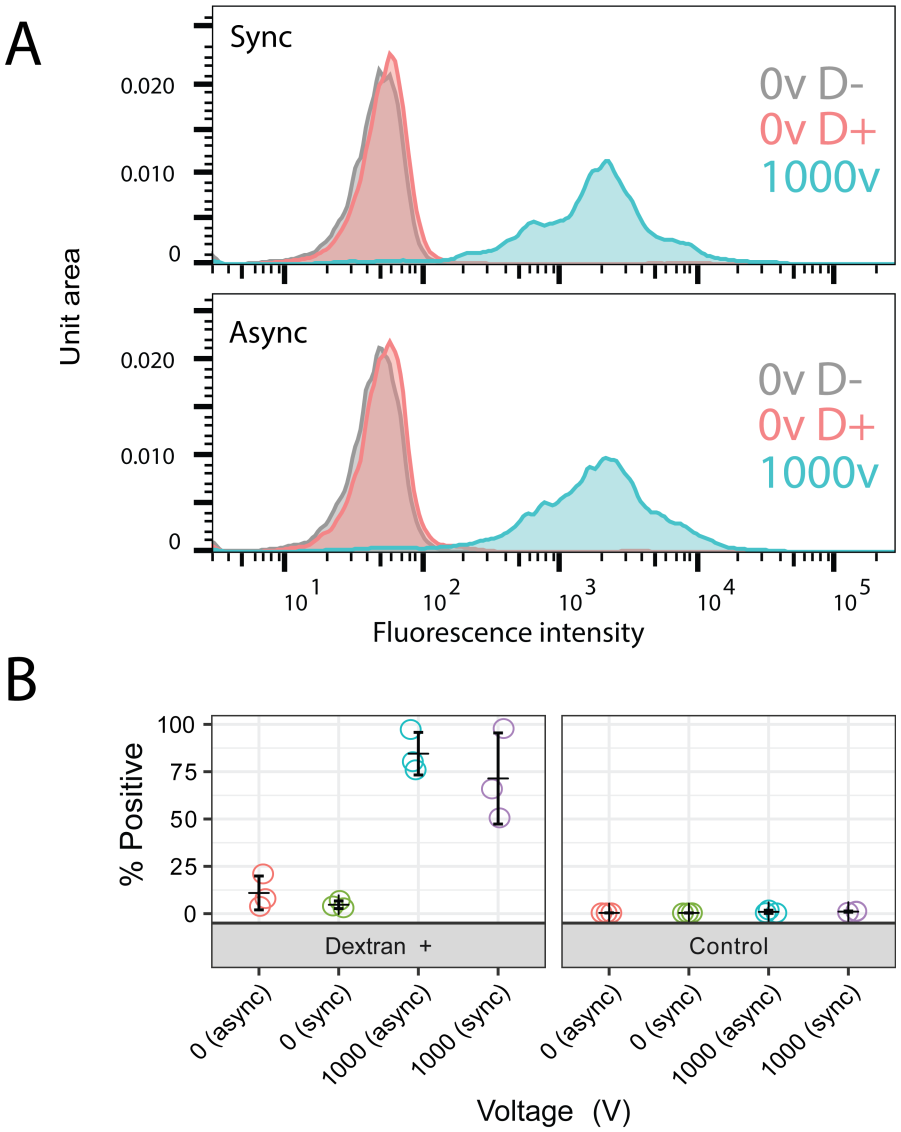

Supplemental Figure 2. Synchronizing spores does not grossly affect protocol efficiency. **(A)** Representative flow cytometry data from a single replicate showing the fluorescence intensity of single cell events for non-electroporated, no dextran controls (*grey*); non-electroporated, dextran incubated controls (*red*); and electroporated, dextrans incubated 1000v treatments (*teal*) for synchronized (sync) and non-synchronized (async) cells. **(B)** Percent of single cell events with fluorescence intensities above the non-electroporated, no dextran control relative to all single cell events for three biological replicates.

B. dendrobatidis Electroporation Protocol Checklist v1.0

Day Before:

- Chill all related media and buffers in 4C except for Bonner's salts

Before Starting:

- [ ] Synchronize cultures 2 hr before collecting cells
- [ ] Calculate the volumes of buffers, media, and dextrans you will need
- [ ] Make fresh SM buffer
    - 50 mL SM:
        - 5 mL 50 mM KCl (autoclaved)
        - 5 mL 150 mM sodium phosphate buffer pH 7.2 (filter sterilized)
            - Alternately, add 750 uL 1M sodium phosphate buffer pH 7.2 (filter sterilized)
        - 7.5 mL 100 mM $MgCl_2$ (filter sterilized)
        - 0.338 g Sodium succinate dibasic acid hexahydrate
        - 0.228 g D-Mannitol
        - milliQ-H_2O to volume (autoclaved)
        - Filter sterilize
- [ ] Make fresh fixation buffer
    - For 5mL PFA fixation:
        - 1.25 mL 16% PFA
        - 2.5 mL water
        - 1.25 mL Cacodylate
            - 1 16% PFA : 2 sterile water : 1 200mM cacodylate
            - Anywhere between 1:1 and 1:4 fixation cells:fixation buffer works to fix them and they look good afterwards.
- [ ] Make dextrans (**be sure it is Invitrogen by thermo fisher scientific cat No. D3305**) at 2 mg/mL in SM
    - Dextrans is sticky, measure in tubes
    - Spin down at high speed to separate insoluables and take supernatant
- [ ] Put SM buffer, dextrans, media, and cuvettes on ice -- allow to cool to 4C
- [ ] turn on centrifuge to 4C
- [ ] Use 2mm Bulldog Bio electroporation cuvettes

Tube Scheme (E = electroporation; D = dextrans):

- E+D+: experimental samples (Cuvette)
- E+D-: accounting for autofluorescence from dead/electrocuted cells (Cuvette)
- E-D+: endocytosis and stickyness (1.5 eppendorf)
- E-D-: buffer happiness and control for the experiments (1.5 eppendorf)

Wash Cells

- [ ] Spin down cells and dump media
- [ ] Resuspend in 10 mL of chilled SM buffer (2500rcf for 5min)
    - If working with multiple tubes, combine during wash
- [ ] Spin down cells and dump SM 2 times (2500rcf for 5min)
- [ ] Resuspend cells in SM buffer to the appropriate concentration & volume
    - 100uL of zoospore suspension for each cuvette.
    - Count concentration, aiming for at least 2x10^7 cells/mL
- [ ] Add 100 uL suspension to each cuvette
    - each cuvette should hold:
        - 100 uL of ~2,000,000 cells (20,000,000 cells/mL concentration)
        - And one of either:
            - 1 Aliquot of Dextrans if D+ (100 uL)
            - 1 Aliquot of SM if D- (100 uL)
    - Total cuvette volume: 200 uL
- [ ] Allow samples to **rest on ice 10 minutes** before electroporating

Electroporation

- [ ] Set electroporation protocol
    - Squarewave protocol (1000V)
    - 3 ms
    - 2 pulses
    - 5 sec intervals
    - 2mm cuvette
- [ ] Insert cuvette
- [ ] Hit the PULSE button, record % droop.
- [ ] Remove cuvette and place on ice. **Allow samples to rest for 10 minutes**
- [ ] Add 200 uL of chilled media to each cuvette and **allow to rest for 10 minutes before washing**

Wash cells

- [ ] 3X with 5 mL chilled sterile (autoclave) growth media
    - 2500 rcf for 5 min
    - Pour off supernatant IMMEDIATELY but gently in one smooth motion without backwash
- [ ] 2X with 5 mL sterile (autoclave) Bonner's/Dilute salts - final resuspension in 600 uL Bonner's/Dilute salts
    - 2500 rcf for 5 min
    - Pour off supernatant IMMEDIATELY but gently in one smooth motion without backwash

[ ] Aliquot 100 uL for counting

[ ] Aliquot 100 uL for imaging

[ ] Fix the remaining cells

- Pipette ~400uL of cells into a 1.5mL eppendorf
- Put equal volume of fixation buffer in each 1.5mL tube of cells
- Fix for 15 min on ice
- Spin 2200 rcf 5 min
- Resuspend in 200 uL Bonner's/Dilute salts
- Either go analyze by flow cytometry or store in TC room fridge for flow at a later date
